# Altruism, Environmental Concerns, and Pro-environmental Behaviors of Urban Residents: A Case Study in a Typical Chinese City

**DOI:** 10.3389/fpsyg.2021.643759

**Published:** 2021-06-07

**Authors:** Ying Xu, Wanxin Li, Shangxin Chi

**Affiliations:** ^1^Department of Sociology, Law School, Shenzhen University, Shenzhen, China; ^2^School of Energy and Environment, City University of Hong Kong, Hong Kong, China; ^3^School of Sociology and Anthropology, Xiamen University, Xiamen, China

**Keywords:** altruism, environmental concerns, environmental behaviors, individual participation, organizational participation, policy participation, China

## Abstract

To investigate the relationships between altruism, environmental concerns, and ordinary people's pro-environmental behaviors that go beyond self-interested NIMBY-ism, we examined measurements of altruism and environmental concerns in a Chinese context and developed a scale that measured people's pro-environmental behaviors at the individual, organizational, and policy level. We then conducted a tailor-made, face-to-face survey (*N* = 603) and found, first, that old age, gender (being a woman), party affiliation, and education level are positively associated with pro-environmental behaviors at the individual, organizational, and policy levels. We next found that human domination worldviews are negatively associated with individual- and organizational-level pro-environmental behaviors and that eco-centric worldviews are positively associated with individual-level pro-environmental behaviors. Third, we found that altruistic behaviors (prosocial behaviors and/or donations) are positively associated with pro-environmental behaviors. In short, awareness of the ecological crisis and altruism can stimulate people's pro-environmental behaviors in China. Meanwhile, it is doubtful that people care more for the environment after their living standards have improved, because socioeconomic status indicators are not statistically significant for individual-level pro-environmental behaviors.

## Introduction

While China's pollution levels continue to increase, people's environmental awareness has also increased over recent decades (Gilley, 2012; Lu et al., 2019). However, scholars have argued that people's environmental concerns may not correspond with their environmental behaviors (Harris, 2006; McGranahan and Tacoli, 2006). In other words, attitudinal questionnaires may be helpful in understanding how respondents believe they will act, but it cannot measure their “true” behavior (Dockery and Bedeian, 1989). For example, although the association between environmental attitudes and behaviors is generally positive (Soutter et al., 2020), empirical studies analyzing the direct relationship between environmental concern and behavior have consistently supported the conclusion that the relationship is low to moderate (e.g., Weigel and Weigel, 1978; Hines et al., 1986/87; Schultz and Oskamp, 1996; Diekmann and Preisendörfer, 1998). Moreover, McGranahan and Tacoli (2006) found a negative correlation between environmental concerns and pro-environmental behaviors among urban dwellers and rural migrants.

From a theoretical standpoint, the literature suggests three major assumptions that can explain people's motivation to engage in pro-environmental behaviors: that such behaviors are performed to benefit oneself (i.e., assumption of egoism), to benefit unfamiliar others (i.e., assumption of altruism), or for the act in itself but not for oneself nor anyone else (i.e., assumption of a moral principle) (e.g., Hardin, 1977; Kahneman and Knetsch, 1992; Batson, 1994; Clark et al., 2003; Hong, 2006; Eom et al., 2019).

Of all three assumptions, the assumption of egoism has been dominant in research on environmental behavior. Many studies have revealed that gaining personal benefits (e.g., better living environment, health, and/or psychological gratification) ranks among the most important factors motivating pro-environmental behaviors (e.g., Kahneman and Knetsch, 1992; Nunes and Schokkaert, 2003; Daube and Ulph, 2016; Hartmann et al., 2017). In particular, of various pro-environmental organizational behaviors, so-called “not in my back yard” (NIMBY) activities in China have drawn great attention from scholars (Ho, 2001; Stalley and Yang, 2006; Lang and Xu, 2013; Wu, 2014; Gu, 2016).

Meanwhile, research has also verified that pro-environmental behaviors have inherent characteristics of altruism (Clark et al., 2003; Griskevicius et al., 2010; Bolderdijk et al., 2013) and pro-environmental principles such as concern for the environment (Dunlap et al., 2000; Hong, 2006; Halkos and Matsiori, 2017; Eom et al., 2019). Thus, self-interested individuals will behave in pro-environmental ways when their behavior benefits them personally but not when the benefit is exclusively environmental. By contrast, altruistic individuals and environmental ethics supporters will engage in pro-environmental behaviors when the benefits are personal and, critically, even if they are solely environmental (Passmore, 1974; De Dominicis et al., 2017).

It is worth noting, for ordinary people who are not involved in NIMBY activities, “The impact of an individual's pro-environmental behavior on its own marginal welfare is rather negligible” (Hartmann et al., 2017: 44). Therefore, ordinary people who engage in pro-environmental behaviors may act for the benefit of the common good (e.g., for future generations) and for the environment itself (e.g., to conserve nature for its own sake) instead of themselves. In that regard, the assumptions of altruism and a moral principle should be important drivers of pro-environmental behaviors. However, in the literature from China, only a few articles discuss the possible effects of altruism and environmental ethics on environmental behaviors at a conceptual level (Yan, 2009; Li, 2016), whereas the possible relationships between altruism, environmental ethics, and environmental behaviors in China have not yet been examined empirically.

This study aimed to fill this research gap. By using a survey developed especially for this study, we adopted the assumption of altruism and the assumption of a moral principle to investigate the pro-environmental behaviors of ordinary people in China that go beyond self-interested NIMBY-ism. More specifically, we sought to:

- Measure a broad range of ordinary people's everyday pro-environmental behaviors;
- Measure altruism and environmental concerns in a Chinese context; and
- Assess how those pro-environmental behavioral patterns correspond to altruism, environmental concerns and demographic factors.

## Literature Review and Hypotheses

### Revisiting Environmental Behaviors in China

People's environmental behaviors have been studied at the individual, organizational, and policy levels in China (Ru, 2004; Yang, 2005; Wang and Lin, 2010). At the individual level, although the specific findings vary, studies have shown that demographic characteristics (e.g., gender, age, and education) significantly impact people's pro-environmental behaviors, including making green purchases and donating to environmental protection organizations (Gong and Lei, 2007; Hong and Xiao, 2007; Chen et al., 2011; Zhang, 2012, 2016). For example, several studies have revealed that women in China express less environmental concern than men (Hong and Xiao, 2007), whereas others have shown that women display greater levels of environmentally friendly behaviors (Gong and Lei, 2007) and that women, older individuals, and people with higher levels of education have greater environmental concern than others (Li, 2003; Luo and Deng, 2008; Shen and Saijo, 2008; Zhang, 2012, 2016). Such controversial results are due to factors of social context. After all, China's borders contain areas with striking differences in not only climate and landscape but also in the people who live there. Such diversity urges researchers to be cautious when choosing instruments for measurement and when making inferences from results.

At the organizational level, Ru (2004) has identified 42 types of activities conducted by Chinese environmental non-profit organizations(ENPOs) (except for the international and student organizations); examples include holding elections, running board meetings, fundraising, interests-based activities (bird watching, tree planting, etc.), organizing exhibitions (such as photograph exhibitions), and street tabling/dissemination of information or souvenirs. Although several well-organized environment-related collective actions in China (e.g., protests against the paraxylene petrochemical plant in Xiamen and incinerators in Beijing, Wujiang, and Panyu) have drawn great attention from scholars (Lang and Xu, 2013; Wu, 2014; Gu, 2016), little research has been conducted to study ordinary people's participation in environmental protection at the organizational level. However, as scholars have revealed, such environmental movements, by taking aim at local projects that tend to prompt short-term concessions and that may prompt polluting companies to merely shift operations toward less-populated and/or investment-hungry provinces, are unlikely to affect environmental protection in China at the policy level (Ho, 2001; Stalley and Yang, 2006; Li et al., 2012).

At the policy level, it is worth noting, regulations of the People's Republic of China on Open Government Information (OGI), which came into effect on May 1, 2008, empowered citizens' public participation at the policy level. According to OGI, officials are required to publicize information about land use, government spending, public health, food and drug safety—anything directly affecting citizens. Thus, by visiting the government or certain ENGOs' official websites, the ordinary people can get various information such as public hearings on environmental assessment, the latest related regulations, and so forth. Nevertheless, to date, studies have primarily focused on students and/or environmental leaders but not the main stream of people in China who have never engaged in any environmental movements. For instance, given the rapid growth of ENPOs, Yang (2005) has proposed that they could serve both as onsite schools and as agents of democratic social change in China. By comparison, Ho (2001) has argued that China's brand of environmentalism has a “female mildness,” meaning going green as long as no conflicts arise and only at a safe distance from direct political action (p. 916). Stalley and Yang's (2006) case study also revealed that altruistic environmentalism among students was unlikely to become an independent movement or a source of pressure for changing policy in China. Beyond that, however, ordinary people's pro-environmental behaviors at the policy level (e.g., to visit the website to collect the related policies, to join a public hearing, etc.) are seldom discussed.

Based on above literature review, to gain a better understanding of the environmental behaviors of ordinary people in China who have never engaged in any environmental movements, we categorized pro-environmental behaviors as occurring at the individual, organizational, or policy level. Likewise, the impacts of demographic factors on the ordinary people's behaviors in relation to the environment were explored at the individual, organizational, and policy levels.

### Altruism and the First Hypothesis: The Assumption of Altruism

In general, altruism can be defined as an individual performing an action which is at a cost to themselves, but benefits, either directly or indirectly, another individual, without the expectation of reciprocity or compensation for that action (Batson, 1987, 2011; Johnson et al., 1989; Eckel and Grossman, 1996; Van Lange et al., 1997). Indeed, debate persists over whether individuals are ever truly altruistic, because people may perform so-called “good deeds” for egoistic reasons, including for public praise or to avoid guilt (Hardin, 1977; Batson, 1987; De Dominicis et al., 2017). Environmental behaviors can also be motivated by reasons of either altruism or self-interest (Guagnano, 2001). However, in research on altruism at the societal level, investigating observable behaviors is more appropriate than investigating internal motivations (Comte, 1875).

Altruism may also be a universal virtue across all societies (Johnson et al., 1989; Simon, 1990; Sober and Wilson, 1998; Madsen et al., 2007; Eom et al., 2019). For instance, in Western thought, Hume (1896, 1902) and Smith (1759) discussed the possibility of human action based on unselfish motives, or what they termed benevolence. Comte (1875), who coined the term altruism, also believed that some social behavior was an expression of an unselfish desire to “live for others” (p. 556). In China, the ancient philosopher Mencius (370–286 BCE) elaborated upon the idea of benevolence and described altruistic actions in various texts that have since circulated for thousands of years:

Everyone has a heart which cannot bear the suffering of others … If anyone sees a child about to fall into a well they will feel fear, not because they may impress the child's parents or their neighbors and friends. From this we can see that compassion is essential to humanity, along with shame, modesty and acceptance (Mencius, 2009 2A:6)

Expend the respect of the aged in one's family to that of other families; expend the love of the young ones in one's family to that of other families (Mencius, 2009 1A:7).

Because pro-environmental behaviors have been shown to positively relate to altruistic values (Clark et al., 2003), we hypothesized that altruism positively relates to environmental behaviors at the individual, the organizational, and the policy level (H1). Our sub-hypotheses were as follows:

H1a: Altruism positively relates to environmental behaviors at the individual level.
H1b: Altruism positively relates to environmental behaviors at the organizational level.
H1c: Altruism positively relates to environmental behaviors at the policy level.

### Environmental Concerns and the Second Hypothesis: The Assumption of a Moral Principle

The assumption of a moral principle, which suggests that environmental behaviors involve moral judgment, partly adheres to the Kantian rule of the supreme principle of morality, that “neither fear nor inclination to the law is the incentive which can give a moral worth to action; only respect for it can do so” (Kant, 1959, p. 440). Similarly, Xunzi (“Master Xun”), a renowned philosopher of China's Warring States Period (481–221 BCE), in his chapter titled “Discourse on Nature” states that nature acts as it always does and that its processes do not change from one epoch to the next (Goldin, 2005).

Considering the assumption of a moral principle, scholars have studied people's environmental attitudes and affirmed that environmental concern—that is, an attitude of seriousness toward environmental problems (Attfield, 1983; Benson, 2001; Dunlap and Jones, 2002)—may affect various pro-environmental behaviors, including responsible consumption and supporting environmentally friendly policies (Nisbet and Myers, 2007; Halkos and Matsiori, 2017; Hall et al., 2018; Eom et al., 2019). For example, environmentalists advocate banning waste incineration for energy, not because pollution occurs in their neighborhoods but because they believe that people in most cities are exposed to toxic concentrations of nitrogen dioxide and particulates, among other pollutants.

Thus, we hypothesized that environmental concern positively relates to environmental behaviors at the individual, organizational, and policy levels (H2). Our sub-hypotheses were as follows:

H2a: Environmental concern positively relates to environmental behaviors at the individual level.

H2b: Environmental concern positively relates to environmental behaviors at the organizational level.

H2c: Environmental concern positively relates to environmental behaviors at the policy level.

## Research Design

Because altruism is a virtue encouraged by various cultures (Johnson et al., 1989; Simon, 1990; Sober and Wilson, 1998; Madsen et al., 2007), researchers need to consider how differences in sociocultural factors, including language, social norms, and social structures, can impact the conduct and interpretation of their empirical research (Allison, 1992; Shiu-Thornton, 2003; Eom et al., 2019). Therefore, in this section, we carefully justify our selection of research setting, target population, and sample, as well as measurements for altruism, environmental concern, and environmental behaviors.

### The Research Site: A Typical Chinese City

Led by the research objectives, we conducted a survey (*n* = 603) from May 9 to May 27, 2014, in a typical Chinese city, which is a cradle of traditional Chinese civilization. According to the city's bureau of statistics, the registered population (i.e., the *hukou* of the City) was 6,999,900 at the end of 2013. In contrast to the metropoles of Beijing, Hong Kong, and Shanghai, this city is more representative of all Chinese cities in its institutional system, size, and culture. To ensure the anonymization, we use “the city” or “the typical city” instead of the city's real name in this article.

The city is located at the juncture of low mountains and hills, such that its terrain is high in the south and low in the north. Dust and pollutants descend upon the city when the wind blows from the north, and due to air pollution, children in the city have often had to stay inside for physical exercise in the winter.

The city's environmental transparency, which can raise public awareness of environmental issues and give the public the tools that it needs to identify and mitigate environmental risks, improved in the 3 years before we conducted our survey. According to the Pollution Information Transparency Index (PITI), which aims to assess the disclosure of sources of pollution-related information, identify and promote good local practices, and encourage the disclosure of information about the environment in general, the city ranked 74th among 113 cities in China in 2011, 61st among 113 cities in 2012, and 24th among 120 cities in 2013 (IPE and NRDC, 2012, 2014).

For the above reasons, the research site, as a typical city in China that faces stress due to environmental problems and has begun to make efforts to reduce pollution, deserves an investigation.

### Research Ethics

This study was approved by the Survey and Behavioral Research Ethics Committee. We prepared and distributed the “Consent Form for an Anonymous Survey” that provided enough information for prospective respondents to decide whether or not to take the survey. The form briefly introduces the study, states that participating in the research is voluntary and that respondents are free to refuse to participate and may withdraw from the research at any time, states that the data would be anonymized and used only for academic purposes, and provides the principal investigator's name and contact information.

### Pilot Study

A pilot study was conducted from November 28 to December 5, 2013. Thirty questionnaires were collected in the aforementioned city to test the reliability of the Chinese Self-Report Altruism Scale (SRA, by Rushton et al., 1981) which was translated by us and New Ecological Paradigm Scale (NEP, by Dunlap et al., 2000) which was translated by Hong (2006). We applied IBM SPSS Statistics V22.0 to analyze the data. And found those values were acceptable (Cronbach's α > 0.7). Once several items were edited to correct minor grammatical errors, the questionnaire was finalized.

### Sampling and Data Collection

To ascertain the main patterns of ordinary people's environmental behaviors, we combined random sampling and purposive sampling to collect data in the city. The sampling procedure was conducted as follows.

First, we randomly selected three districts from among the six districts in the city. Next, we randomly selected a street office (*jiedaobanshichu*) in each district. Each sub-district is in charge of ~10 neighborhoods (*Juweihui*), and the total population managed by a street office exceeds 50,000. At that point, in each sub-district street office, we randomly selected a neighborhood where no NIMBY activities had occurred in the previous 3 years. Although random sampling can be highly representative of the population, to avoid possible residential segregation and ensure that participants sampled represented variety in social background, we selected one high-end residential estate and one inexpensive residential estate in each neighborhood. Altogether, six estates were included in the study.

Next, we hired 12 field interviewers to conduct a face-to-face survey. The interviewers knocked on the doors with odd room numbers in the selected estates. People above 18 years old who opened the door were invited to answer the face-to-face questionnaire.

In total, 639 questionnaires were distributed, and 603 valid questionnaires were collected. It should be noted, we regret the unexpected missing data. This may be due to the mailing distance, which led to some answers being difficult to read. For example, there are 20 items of the SRA scale, however, if one item of the scale is missing, the information of the whole scale is missing. Therefore, there are only 325 valid samples after factor analysis of SRA. In regression analysis, due to the increase of variables, the number of samples will continue to decrease, resulting in the minimum sample size of 216.

### Measuring Altruism, Environmental Concern, and Environmental Behaviors

#### Altruism: Revised Self-Report Altruism Scale

A number of measures—such as prosocial values, social responsibility, moral judgment, and empathy—appear to be stable traits of altruism (Staub, 1974; Rest, 1979; Rushton et al., 1981; Ma, 2013). Scholars have designed a number of scales to measure altruism in various social contexts (Heist and Yonge, 1962; Mehrabian and Epstein, 1972; Ma and Leung, 1991; Khanna et al., 1993; Chou, 1996). Among the direct measurements of altruism, the SRA, developed by scholars in Canada (Rushton et al., 1981), is one of the most widely used scales, even in non-Western societies. For example, Khanna et al. (1993) modified the original SRA to develop a Hindi version of the scale.

Given that the Chinese language, culture, and social context can vary greatly from those in other countries, several scholars have translated and revised the SRA for research in China. For example, Chou (1996) translated the Hindi SRA into Traditional Chinese to assess the altruistic behaviors of adolescents in Hong Kong. More recently, Song and Chen (2012) and Tang et al. (2015) translated the original SRA into Simplified Chinese and revised the scale to measure altruism among college students in mainland China. However, because most people living in mainland China do not read Traditional Chinese and because our research's purposes required a sample including adults from all walks of life and with various levels of education, levels of income, ages, and occupations, none of the original or derived instruments were suitable for our research.

Thus, to confirm factors describing altruism in mainland China, we critically examined the Canadian and Hindi SRAs in light of the Chinese context. In turn, the first author translated both instruments into Simplified Chinese, and the translations were assessed by a professor of English linguistics whose native language is Mandarin. After that, we invited an expert in social psychology and an expert in social research methods to comment on the items of the Canadian and the Hindi SRA scales. The two experts agreed that both scales would need to be revised before they could be applied in China. At that point, we recruited 77 full-time postgraduate students majoring in social sciences who hailed from 31 provinces of China. The students were randomly divided into group A (38 students) and group B (39 students). Group A was asked to respond to the Canadian SRA, and group B was asked to respond to the Hindi SRA. At the same time, the students were asked to comment on each item of the scale by answering the following questions: Does the translation of the item match Chinese expressions/usage? Does the content of the item correspond to Chinese culture or social reality? Do you have any other opinions?

The opinions about the Canadian SRA included the following ideas. First, the situation of “I have helped push a stranger's car out of the snow” would seldom occur in many parts of China. Second, regarding “I have given a stranger a lift in my car,” more than 70% of the students expressed that due to the worry of endangering their own safety, people would not give a ride to strangers, and they believed that other Chinese people would not do so either. They stated that this question may not reflect altruism well, given the low level of social trust in China. At the same time, three students said that they had given strangers a lift, two students had been given a lift by strangers, and 14 students stated that they might give others a lift.

The opinions about the Hindi SRA included the following ideas. First, the “scooter” or “motorbike” (frequently used in this scale) is not the major means of transportation in China and would need to be revised. Second, the use of assumed imagination in the scale should be discussed, as imagined behaviors may be different from behaviors in reality. Furthermore, self-enhancing bias is likely to occur when someone is invited to report their altruistic attitude, although the “imagined activities” and the real acts may be linked (Gosling et al., 1998; Baumeister, 1999).

Therefore, in line with the opinions collected, a Chinese SRA was developed by slightly modifying the Canadian SRA. After much deliberation, the item, “I have helped push a stranger's car out of the snow” in the Canadian SRA was replaced with “I have helped a stranger put his/her luggage in the luggage rack” in the Chinese version. This is for two reasons. First, pushing a car and putting luggage in a luggage rack are similar: both require a certain level of physical strength to help others. Second, most public transportation in China (such as planes, cars, trains, etc.) have a luggage rack, so it is more likely that most Chinese people will encounter someone who needs to put luggage on a luggage rack. Additionally, given the lack of consensus regarding the item, “I have given a stranger a lift in my car,” this item was included for further investigation.

The specific measurement items of the Chinese version of the SRA are shown in Table 1. Respondents are asked to rate their engagement in the activity in each item on a five-point Likert scale, where “1 = never,” “2 = once,” “3 = more than once,” “4 = often,” and “5 = very often.” A higher score represents a greater number of altruistic acts.

**Table 1:** Factor analysis of altruism.

**Item**	**Factor analysis**				
	**Prosocial behavior**	**Sympathetic behavior**	**Social responsibility**	**Social donation**	**Communalities**
1. I have helped a stranger to put his/her luggage in the luggage rack.	**0.52**	−0.10	0.26	0.46	0.55
3. I have made change for a stranger.	**0.63**	0.10	0.17	0.28	0.50
5. I have given money to a stranger who needed it (or asked me for it).	**0.63**	0.07	0.02	0.18	0.44
11. I have allowed someone to go ahead of me in a line up (at photocopy machine, in the supermarket).	**0.54**	0.33	0.14	−0.21	0.47
17. I have, before being asked, voluntarily looked after a neighbor's pets or children without being paid for it.	**0.59**	0.28	0.13	0.22	0.48
18. I have offered to help a handicapped or elderly stranger across a street.	**0.47**	0.26	0.26	0.32	0.46
20. I have helped an acquaintance to move households.	**0.64**	0.13	0.28	0.13	0.51
7. I have done volunteer work for a charity.	0.10	**0.69**	0.02	0.32	0.59
8. I have donated blood.	0.37	**0.42**	−0.22	0.21	0.41
12. I have given a stranger a lift in my car.	0.52	**0.53**	−0.10	−0.20	0.61
15. I have bought “charity” Christmas cards deliberately because I knew it was a good cause.	0.16	**0.72**	0.07	0.16	0.57
16. I have helped a classmate who I did not know that well with a homework assignment when my knowledge was greater than his or hers.	0.10	**0.62**	0.30	0.12	0.50
2. I have given directions to a stranger.	0.05	−0.07	**0.66**	0.41	0.61
10. I have delayed an elevator and held the door open for a stranger.	0.22	0.13	**0.74**	0.03	0.61
13.I have pointed out a clerk's error (in a bank, at the supermarket) in undercharging me for an item.	0.16	0.47	**0.48**	−0.11	0.49
19. I have offered my seat on a bus or train to a stranger who was standing.	0.14	0.06	**0.80**	0.02	0.66
4. I have given money to a charity.	0.27	0.23	0.16	**0.68**	0.61
6. I have donated goods or clothes to a charity.	0.22	0.40	−0.06	**0.67**	0.65
Eigenvalues	2.99	2.58	2.31	1.83	9.71
Percentage of variance	16.62	14.34	12.81	10.19	53.96

*The bold values in one column means that the items load onto one factor*.

We adopted principal components analysis (PCA) to analyze the 20 items of the Chinese SRA. Factors with eigenvalues greater than one are subjected to a varimax rotation. It was found that two items had communalities of <0.3 (9 - “I have helped carry a stranger's belongings [books, parcels, etc.]” and 14 - “I have let a neighbor whom I didn't know well borrow an item of some value from me [e.g., a dish, tools, etc.]”). We excluded items 9 and 14, and then applied PCA to the 18 items of the scale. As the results shown in Table 1, seven items load most heavily on the first factor, which we named “prosocial behaviors” factor (1, 3, 5, 11, 17, 18, 20). It is not only because the items described the prosocial activities, but also because the relationship between altruism and prosocial attitude has been long discussed (Schwartz, 1972; Batson, 1987). Five items load most heavily on the second factor, which we named the “sympathetic behavior” factor (7, 8, 12, 15, 16). This is because these items embody sympathetic elements, and it was believed that sympathy could be one of the altruistic motivation (Krebs, 1975). Four items load most heavily on the third factor, which we named the “social responsibility” factor (2, 10, 13, 19). Though there is a debate that engaged social responsibility not always being driven by altruism, scholars have suggested certain links between social responsibility and altruism (Tang et al., 2015). Two items load most heavily on the fourth factor, which we named the “social donation” factor (4, 6). As one of the most popular altruism behaviors in various cultures, donation could be an important dimension of altruism (Eckel and Grossman, 1996). The Cronbach's alpha coefficient of the above mentioned factors were 0.78, 0.70, 0.71, and 0.50, respectively.

As we mentioned earlier, though a few studies have used the SRA scale, there is no consensus on the measurement in terms of translations and dimensions among Chinese scholars (Chou, 1996; Song and Chen, 2012; Tang et al., 2015). To facilitate a more direct analysis of the effect of each independent variable on the dependent variable in the regression model, we used a formula to convert these factors into an index between 1 and 100 (Table 2). Thus, the *mean* of people's altruism in prosocial behavior, sympathetic behavior, social responsibility, and social donation of participants engaging in pro-environmental behaviors are 56.46, 41.20, 62.51, and 51.68, respectively (Table 2).

**Table 2:** Descriptive statistics of research variables

**Continuous variable**	**Mean**	**Standard deviation**	**Sample size**	**Category variable**	**Percent %**	**Sample size**
**Dependent variable:** *Pro- Environmental behaviors*				**Age**	100	531
Individual participation factor	55.59	20.91	483	Young adults (18–44)	55.18	293
Organizational participation factor	13.95	15.30	483	Middle adults (45–59)	20.15	107
Policy participation factor	36.95	21.21	483	The elderly (60+)	24.67	131
**Independent variable:** *Environmental Concerns*.				**Political status**	100	500
Human domination factor	55.63	13.68	544	CPC Member	23.60	118
Eco-crisis factor	59.09	17.12	544	Non-CPC Member	76.40	382
Balance of natural factor	57.21	18.35	544	**Education level**	100	567
**Independent variable:** *Altruism*				Junior Secondary and Below	25.75	146
Prosocial behavior factor	56.46	18.25	325	Senior Secondary	25.93	147
Sympathetic behavior factor	41.20	18.20	325	College or University	44.78	254
Social responsibility factor	62.51	19.15	325	Postgraduate and above	3.53	20
Social donation factor	51.68	16.76	325	**Personal monthly income**	100	526
**Category variable**	Percent %	Sample size		0–1,999¥	34.03	179
**Gender**	100	558		2,000–3,999¥	46.01	242
Male	43.55	243		4,000–5,999¥	11.79	62
Female	56.45	315		6,000–7,999¥	4.37	23
				≥8,000¥	3.80	20

*We used the following formula to convert these factors into an index between 1 and 100:*.

*Convert factor = (factor + B) • A*.

*A = 99/(maximum factor- minimum factor)*.

*B = (1/A) - minimum factor*.

#### Environmental Concern: A Chinese Version of the New Environmental Paradigm

Chinese scholars have translated major scales, including the New Environmental Paradigm (Dunlap and Van Liere, 1978), the Environmental Concern Scale (Schultz and Zelezny, 1998, 1999), and the NEP (Dunlap et al., 2000) and suggested modifying them to better suit Chinese culture and society (Chung and Poon, 1999; Hong, 2006; Xiao and Hong, 2007; Liu and Wu, 2012; Hong et al., 2014). However, no consensus on the measurement of environmental concern in China has been reached. Some scholars have adopted a translated version of the original scale, whereas others have deleted several items, and still others have introduced new items (Luo and Deng, 2008; Duan, 2009; Luo et al., 2009; Feng, 2010; Zhou, 2011).

On the basis of a pilot study, we adopted the NEP scale (Dunlap et al., 2000) translated by Xiao and Hong (2007) for three reasons. First, Hong (2006) and several other scholars adopted the translated Chinese version of the NEP in the nationwide Chinese General Social Survey (CGSS) in 2003 and 2010, and they acknowledged that the scale can be an important instrument for measuring the general public's environmental attitudes when properly altered (Xiao and Hong, 2007; Hong et al., 2014). Second, because no consensus exists on the revision of the NEP scale, applying the original NEP (i.e., the Chinese version) remains the most appropriate option. Beyond that, as Hong et al. (2014) have suggested, the scale can be further revised by deleting certain items with low resolution coefficients in data analysis. Third, because the NEP has become a widely used measure in pro-environmental research in more than 40 countries (Hawcroft and Milfont, 2010), using the scale may improve dialogue between research and benefit knowledge accumulation.

The specific measurement items of NEP are shown in Table 3; the answer options consist of five items, including “1 - strongly disagree,” “2 – disagree,” “3 – undefined,” “4 – agree,” and “5 - strongly agree.” A higher score represents a greater degree of environmental concern^1^.

**Table 3:** Factor analysis of environmental concerns.

**Item**	**Factor analysis**			
	**Human domination**	**Eco-crisis**	**Balance of nature**	**Communalities**
2. Humans have the right to modify the natural environmental to suit their needs.	**0.61**	0.04	−0.02	0.37
4. Human beings' ingenuity will ensure that we do NOT make the earth unlivable.	**0.56**	−0.05	−0.05	0.32
6. The earth has plenty of natural resources if we just learn how to develop them.	**0.60**	0.18	0.05	0.39
8. The balance of nature is strong enough to cope with the impacts of modern industrial nations.	**0.67**	0.15	0.13	0.49
10. The so-called “ecological crisis” facing humankind has been greatly exaggerated.	**0.55**	0.23	−0.07	0.36
12. Humans beings were meant to rule over the rest of nature.	**0.60**	0.09	0.22	0.41
14. Humans will eventually learn enough about how nature works to be able to control it.	**0.64**	0.03	0.22	0.45
3. When human beings destroy nature, it often produces disastrous consequences.	0.14	**0.65**	0.25	0.50
5. Human beings are abusing and destroying the environment.	0.11	**0.67**	0.20	0.50
7. Plants and animals have as much right as human beings to exist.	0.14	**0.69**	−0.10	0.51
13. The balance of nature is very delicate and easily upset.	0.04	**0.67**	0.23	0.50
15. If things continue on their present course, we will soon experience a major ecological catastrophe.	0.10	**0.58**	0.35	0.47
1. We are approaching the limit of the number of people the Earth can support.	0.06	0.07	**0.77**	0.61
9. Despite our special abilities, human beings are still subject to the laws of nature.	0.11	0.21	**0.58**	0.39
11. The earth is like a spaceship with very limited room and resources.	0.03	0.31	**0.60**	0.46
Eigenvalues	2.61	2.40	1.70	6.70
Percentage of variance	17.42	15.97	11.31	44.69

*The bold values in one column means that the items load onto one factor*.

We adopted PCA to analyze the 15 items measuring environmental concerns. Factors with eigenvalues greater than one are subjected to a varimax rotation. It is worth noting, as Chinese scholars indicated: our sample did not support the five dimensional structure of the NEP (Dunlap et al., 2000). Still, we used the broadly-used terms to name the factors to improve understanding between scholars (Dunlap et al., 2000; Hong, 2006). Of the results shown in Table 3, seven items load most heavily on the first factor, which we named, “Human Domination” factor (2, 4, 6, 8, 10, 12, 14). Five items load most heavily on the first factor, which we named, “Eco-Crisis” factor (3, 5, 7, 13, 15). And three items load most heavily on the first factor, which we named, “Balance of nature” factor (1, 9, 11). Their Cronbach's alpha coefficients were 0.72, 0.73, and 0.50, respectively. The mean of human domination, eco-crisis, and balance of nature were 55.63, 17.12, and 57.21 (Table 2).

#### Environmental Behaviors

With increased awareness of environmental degradation and anxiety over health, evidence has shown that Chinese citizens have begun to participate voluntarily in various pro-environmental activities, including: donating to environmental protection organizations, participating in environmental volunteering, conducting environmental research, establishing environmental organizations or groups, and conducting “not-in-my-backyard” (NIMBY)-style movements such as protesting against pollution (Ho, 2001; Ru, 2004).

On the basis of a comprehensive literature review and previous research (Kaiser, 1998; Kaiser et al., 2003; Ru, 2004), we developed 20 questions to study the frequency and forms of pro-environmental behaviors, which covered the *individual* level (e.g., environmental consumption), the *organizational* level (e.g., establishing environmental organizations) and the *policy* level (e.g., make suggestions on environmental policies to the government). The specific measurement items are shown in Table 4: the answers are either “yes” or “no,” with values of 1 and 0, respectively. PCA was used to analyze the nine items measuring environmental behaviors. We employed varimax rotation to create orthogonal dimensions.

**Table 4:** Factor analysis of environmental behaviors.

**Item**	**Factor analysis**			
	**Policy participation**	**Individual participation**	**Organizational participation**	**Communalities**
7. Have you ever heard of the “environmental disclosure rules”?	**0.73**	0.07	0.05	0.54
8. Have you taken part in an environmental public hearing?	**0.65**	0.02	0.32	0.53
9. Do you know that the government has solicited public opinions on environmental protection?	**0.66**	0.16	−0.15	0.48
1. Do you have the habit of bringing your own shopping bags?	0.11	**0.62**	0.06	0.40
2. Do you sort your waste?	0.09	**0.71**	0.01	0.51
3. Do you buy phosphorus-free detergent?	−0.01	**0.62**	−0.08	0.39
4. Have you done any volunteer work relating to environmental protection?	0.23	**0.40**	0.32	0.32
5. Have you initiated/organized environmental protection activities?	−0.05	−0.01	**0.82**	0.67
6. Have you ever founded an environmental protection organization?	0.11	0.02	**0.72**	0.52
Eigenvalues	1.5	1.5	1.4	4.4
Percentage variance	16.4	16.3	15.7	48.4

*The bold values in one column means that the items load onto one factor*.

As the results shown in Table 4, the three factors with eigenvalues greater than one are subjected to a varimax rotation. Three items loading most heavily on the “policy participation” factor, consist of items (7, 8, 9) designed to tap the facet of pro-environmental behaviors at the policy level. Four items loading most heavily on the “individual participation” factor, consisted of items (1, 2, 3, 4), which were designed to tap the facet of pro-environmental behaviors at the individual level. It is worth noting, the formal volunteering, in general, is a kind of “organized volunteering,” organized by various organizations (Xu and Ngai, 2011; Xu, 2013). In this regard, the volunteer participation could be categorized into the action at the “organizational level.” However, clearly, the notion of volunteering embodies a set of values, such as altruism that emphasize an action taken by personal choice and without expectation of pay (Dunn, 1995; Xu, 2017). Seen in this light, volunteering can also be regarded as a behavior at the “individual level,” because it is a personal choice based on free will and it is not for remuneration. Of the remaining two items (5, 6), loading was most heavily on the “organizational participation” factor, which were designed to tap the facet of pro-environmental behaviors at the organizational level. The Cronbach's coefficients of the three factors were 0.65, 0.55, and 0.71, respectively.

The mean of individual participation, policy participation, and organizational participation of participants engaging in pro-environmental behaviors were 55.59, 36.95, and 13.95, respectively (Table 2). This indicates that individual participation was highest, followed by policy participation and organization participation.

### Variables and Method

People's pro-environmental behavior is the dependent variable. Independent variables include altruistic behaviors and environmental concerns.

Information on demographic and socioeconomic variables—such as gender, age, political status, education, income, and hometown—was also collected in this study. Demographic variables were used as control variables. These included gender, age, political status, education level, and personal monthly income.

The dependent variables in this study are continuous variables. Thus, a multiple linear regression Model was adopted. And the formula is as follows:

(1) $$\begin{array}{r} y=\beta_{0}+\beta_{1}x_{1}+\beta_{2}x_{2}+...+\beta_{j}x_{j}+\mu \end{array}$$

Among them, “*y*” refers to dependent variables, including the individual participation factor, organizational participation factor, and policy participation factor of the pro-environmental behaviors. And “*xj*” represents the j-th independent variables and control variables.

β*j* represents the regression coefficient corresponding to the *j-th* independent variables or control variables. β*0* is a constant term, and μ is a random error term.

To examine the impacts of altruism and environmental concerns on pro-environmental behaviors respectively, a separate regression has been run with altruism vs. environmental concerns. Shown in Table 5, Models A1, B1, and C1 only included demographic variables; Models A2, B2, and C2 included altruism variables on the basis of Model 1; and Models A3, B3, and C3 included environmental concern variables on the basis of Model 1. The dependent variable of Model A was individual participation, the dependent variable of Model B was organizational participation, and the dependent variable of Model C was policy participation. The explanatory power of altruism and environmental concerns to the dependent variables can be obtained by comparing change in R2 values. Finally, Models A4, B4, and C4 were aggregate Models, which included all of the variables mentioned above.

**Table 5:** Environmental concern, altruism, and environmental behaviors.

	**Individual participation**				**Organizational participation**				**Policy participation**			
	**A1**	**A2**	**A3**	**A4**	**B1**	**B2**	**B3**	**B4**	**C1**	**C2**	**C3**	**C4**
**Gender^a^**	−7.81^***^	−9.20^***^	−7.77^***^	−8.70^***^	1.78	3.34	0.79	2.04	−0.55	−1.52	−0.68	−2.56
	(2.60)	(3.28)	(2.65)	(3.31)	(1.89)	(2.84)	(1.93)	(2.90)	(2.83)	(3.32)	(2.91)	(3.44)
**Age^b^**												
Young adults	−9.31^***^	−10.18^**^	−9.63^***^	−7.44	6.12^**^	6.56	6.29^**^	8.13^*^	−7.13^*^	2.33	−4.50	3.12
	(3.42)	(4.98)	(3.56)	(5.24)	(2.46)	(4.29)	(2.56)	(4.54)	(3.75)	(5.03)	(3.94)	(5.45)
Middle adults	−6.93^*^	−9.61	−5.47	−5.88	1.16	−1.22	1.10	0.43	−7.37^*^	−2.88	−4.17	−0.40
	(3.91)	(5.86)	(4.00)	(6.04)	(2.78)	(5.06)	(2.87)	(5.28)	(4.17)	(5.83)	(4.34)	(6.22)
**Political status^c^**	7.34^**^	8.23^**^	6.50^**^	8.79^**^	2.25	0.05	2.33	0.76	11.90^***^	6.95^*^	12.09^***^	9.00^**^
	(3.13)	(4.11)	(3.20)	(4.22)	(2.25)	(3.57)	(2.30)	(3.69)	(3.47)	(4.19)	(3.59)	(4.40)
**Education level^d^**												
Senior secondary	7.46^**^	10.60^*^	6.00	11.32^**^	1.69	0.17	1.40	−0.33	−0.47	0.01	−2.40	−0.94
	(3.66)	(5.58)	(3.77)	(5.70)	(2.62)	(4.84)	(2.71)	(5.04)	(3.97)	(5.62)	(4.14)	(6.00)
College or university	3.50	1.77	3.76	4.15	−1.42	−7.31	−0.65	−6.11	−2.17	2.99	−2.51	3.16
	(3.62)	(5.32)	(3.74)	(5.43)	(2.57)	(4.65)	(2.67)	(4.82)	(3.94)	(5.39)	(4.13)	(5.70)
Postgraduate and above	8.62	13.20	7.30	13.67	1.14	−4.32	1.23	−4.23	3.60	11.90	1.74	11.09
	(7.81)	(9.01)	(7.83)	(8.93)	(5.76)	(8.04)	(5.78)	(8.09)	(8.67)	(9.22)	(8.77)	(9.45)
**Personal monthly income**	1.20	0.76	1.52	0.94	−1.74^*^	−0.72	−1.50	−0.55	−2.82^*^	−1.43	−2.71	−2.30
	(1.45)	(1.90)	(1.50)	(1.94)	(1.05)	(1.66)	(1.09)	(1.70)	(1.67)	(2.01)	(1.75)	(2.08)
**Altruism**												
Prosocial behavior		0.38^***^		0.33^***^		0.07		0.05		0.19^**^		0.18^*^
		(0.09)		(0.09)		(0.08)		(0.08)		(0.09)		(0.10)
Sympathetic behavior		−0.08		−0.11		0.03		−0.05		0.16		0.11
		(0.09)		(0.10)		(0.08)		(0.09)		(0.09)		(0.10)
Social responsibility		0.19^**^		0.12		0.10		0.10		−0.00		−0.03
		(0.08)		(0.09)		(0.07)		(0.08)		(0.08)		(0.09)
Social donation		0.36^***^		0.29^***^		0.29^***^		0.30^***^		0.22^**^		0.22^**^
		(0.09)		(0.09)		(0.08)		(0.08)		(0.10)		(0.10)
**Environmental concerns**												
Human domination			−0.12^*^	−0.18^*^			−0.09^*^	−0.15^*^			−0.11	0.04
			(0.07)	(0.09)			(0.05)	(0.08)			(0.08)	(0.10)
Eco-crisis			0.22^***^	0.27^**^			0.08	0.04			0.11	0.03
			(0.08)	(0.10)			(0.05)	(0.09)			(0.08)	(0.11)
Balance of nature			0.16^*^	0.18			0.08	0.05			0.12	0.14
			(0.09)	(0.12)			(0.07)	(0.10)			(0.10)	(0.12)
**Constant**	55.31^***^	6.59	39.30^***^	−0.85	8.69^***^	−14.69^*^	3.87	−8.38	34.96^***^	−8.21	26.86^***^	−14.76
	(3.81)	(9.81)	(8.14)	(12.71)	(2.77)	(8.67)	(5.89)	(11.25)	(4.16)	(9.91)	(8.86)	(13.25)
N	410	242	388	229	407	237	384	224	399	230	376	216
F	3.723	5.349	3.900	4.770	1.689	2.236	1.600	2.045	2.890	2.017	2.335	1.693
*R*^2^	0.08	0.20	0.10	0.25	0.03	0.11	0.05	0.13	0.06	0.10	0.07	0.11
Adjusted *R*^2^	0.05	0.16	0.08	0.20	0.02	0.06	0.02	0.07	0.02	0.05	0.04	0.05
Change in *R*^2^	——	0.12^***^	0.02^*^	0.17^***^	——	0.08^***^	0.02^*^	0.10^***^	——	0.04^**^	0.01	0.05^**^

*(1) The coefficient is a non-standardized regression coefficient, with a standard error in brackets*.

*(2)*

a *represents “woman,”*

b *represents “elderly adult,”*

c *represents “non-CPC member,”*

d *represents “junior secondary or below.”*

*(3)*

*** *p < 0.01*,

** *p < 0.05*,

* *p < 0.1*.

*(4) change in R^2^ refers to the change in R^2^ compared to the baseline Model*.

*(5) Significant figures shown in bold*.

## Results and Discussion

As mentioned above, this study applied the multiple linear regression Model by taking the individual-level, the organizational-level, and the policy-level pro-environmental participation as the dependent variables; the dimensions of environmental concerns and altruism as the main predictor variables; and gender, age, political status, education level, and personal monthly income as the control variables. The descriptive statistics of the variables in this study are shown in Table 2. Through collinearity statistics, obtained VIF value of 1–2, it can be concluded that there is no multicollinearity symptoms. The regression results are shown in Table 5.

In terms of individual-level pro-environmental behaviors, gender, age, political status, and education level had a significant impact on individual pro-environmental behaviors. As shown in Model A1, the score of male participants in individual pro-environmental behaviors was lower than that of women, by a difference of 7.81. The scores of young adults and middle-aged participation in individual pro-environmental behaviors were lower than that of older adults, by differences of 9.31 and 6.93, respectively. Members of the Communist Party of China (CPC) displayed more participation in individual pro-environmental behaviors than party non-members. The score of participants who had achieved senior secondary school education was higher than that of participants who had achieved junior secondary school education or below in individual pro-environmental behaviors, by a difference of 7.46. No statistically significant difference was found for the indicator of socioeconomic status, measured by personal monthly income.

In terms of altruism and individual pro-environmental behaviors, the prosocial behavior and social donation factors had a significant positive impact on individual pro-environmental behaviors, and the sympathetic behavior and social responsibility factors had no statistically significant impact on individual pro-environmental behaviors. Compared with Model A1, *R*^2^ of Model A2 increased by 12%, indicating that altruism had a good explanatory power for individual participation. In Model A2, when the scores of prosocial behavior, social responsibility, and social donation increased by 1, the scores of individual pro-environmental behaviors increased by 0.38, 0.19, and by 0.36, respectively. In this regard, altruistic behaviors can promote the pro-environmental behaviors at the individual level. **H1a hypothesis is therefore supported**. However, as Model A4 shown, when the environmental concern variables were included, the effect of social responsibility dimension is non-significant, while the effect of other dimensions remains unchanged.

In terms of the relationship between environmental concern and individual pro-environmental behaviors, the factors of human domination, eco-crisis, and balance of nature were significant in the opposite direction; that is, the anthropocentric factor had a negative effect on individual participation in pro-environmental behaviors. In Model A3, if the score of the human domination factor increased by 1, the score of individual engagement of participants in pro-environmental behaviors decreased by 0.12. The eco-crisis factor and the balance of nature factor had positive effects on individual pro-environmental behaviors. In Model A3, when the scores of the ecological crisis factor and the natural balance factor increased by 1, the scores of individual pro-environmental behaviors increased by 0.22 and by 0.16, respectively. Residents who scored higher on the eco-crisis factors and/or the balance of nature factors engage in more individual pro-environmental behaviors. In other words, human domination is a negative dimension on environmental concern, while the eco-crisis factor and the balance of nature factor are positive dimensions. Thus, in general, environmental concern has a positive effect on pro-environmental behaviors at the individual level. Therefore, **Hypothesis H2a is verified**. However, as Model A4 has shown, when the altruism variables were included, balance of nature dimension becomes non-significant, while the effect of other dimensions remains unchanged. In addition, comparing with Model A1, *R*^2^ of Model A3 increased by 2% only. This indicated that the explanatory power of environmental concerns on individual participation was less than altruism.

In terms of organizational pro-environmental behaviors, the young adults engaged in more pro-environmental behaviors than older people: in Model B1, for example, the score of participation in environmental organizations of the young adults was higher than 6.12. This finding is precisely the opposite of individual environmental behaviors. It can be seen that different age groups have significant differences in their pro-environmental behaviors. Furthermore, personal monthly income had a negative effect on participants' organizational pro-environmental behaviors. Other control variables were not statistically significant in their impact on participation in organizational environmental behavior.

In terms of altruism and organizational-level pro-environmental behaviors, the social donation factor had a significant positive effect on participation in environmental protection organizations. Compared with Model B1, *R*^2^ of Model B2 increased by 8%. This indicated that altruism had a good explanatory power for organizational participation. In Model B2, when the score of the social donation factor increased by 1, the score of organizational participation increased by 0.29. The effect is still significant when adding environmental concern variables. **Therefore, hypothesis 1b is partially verified**. The relationships between the other dimensions and organizational-level pro-environmental participation were not statistically significant.

In the context of environmental concerns and organizational-level pro-environmental behaviors, the human domination factor had a significant negative impact on organizational-level pro-environmental behaviors. Compared with model B1, the R^2^ of model B3 increased by 2%. This indicated that the explanatory power of environmental concerns on organizational participation was less than altruism. In Model B3, if the score of the human domination factor increased by 1, the score of residents' organizational-level pro-environmental participation decreased by 0.09. Thus, people with a human domination consciousness will not only negatively influence their individual participation in pro-environmental behaviors but will also reduce their organizational participations. The effect is still significant when adding altruism variables. **Therefore, hypothesis H2b is partially verified**. The other two dimensions were not statistically significant for organizational participation.

In terms of policy-level pro-environmental behaviors, age, political status, and personal monthly income had a significant impact. Young adults and middle-aged groups showed less participation in policy-level pro-environmental behaviors than the elderly group: for example, in Model C1, the young adults and middle-aged groups had lower scores than elderly people by differences of 7.37 and 7.13, respectively. Party members had a policy-level pro-environmental behavior score that was higher than that of non-party members by 11.9. As can be seen, party members are more actively involved in the revision of environmental protection policies. Like the effect on organizational pro-environmental behaviors, personal monthly income had a negative effect on participants' policy-level pro-environmental behaviors. The relationships between other control variables and participants' policy-level pro-environmental behaviors were not statistically significant.

In terms of altruism and policy-level pro-environmental participation, similar with the individual-level pro-environmental behaviors, the prosocial factor and social donation factor had a significant positive effect on participation in environmental protection organizations. Compared with Model C1, *R*^2^ of Model C2 increases by 4%. This indicated altruism had low explanatory power on policy participation. In Model C2, when the score of the prosocial factor and social donation factor increased by 1, the score of organizational participation increased by 0.19 and 0.22. The effect is still significant when adding environmental concern variables. **Therefore, hypothesis H1c is partially verified**.

In terms of environmental concerns and pro-environmental participation in policy, it was found that all dimensions of environmental concern had no statistically significant effect on policy-level pro-environmental behaviors. It can be seen that environmental concerns mainly affected individual and organizational pro-environmental participation, not policy participation. Therefore, hypothesis H2c has not been supported.

## Conclusions and Further Research

### Summary and Implications

To investigate the relationships between altruism, environmental concerns, and ordinary people's pro-environmental behaviors performed in consideration of how their actions might affect others and that exceeds self-interested NIMBY-ism, we analyzed Chinese-language versions of the SRA and NEP scales before developing our own scale to measure a relatively broad range of people's everyday pro-environmental behaviors at the individual, organizational, and policy level in a Chinese context. Then, by using a tailor-made survey (*n* = 603), we explored the factors that affect ordinary people's pro-environmental behaviors by analyzing the relationships between environmental concerns, altruism, and pro-environmental participation at the individual, the organizational, and the policy levels. The main findings are summarized below.

From a demographic perspective, we found that age, gender, and political status significantly impact people's pro-environmental behaviors. First, in contrast to men and the young adults, women and the elderly are more likely to participate in pro-environmental activities at the individual level. This finding is similar to other studies, which suggests that it may be related to the fact that women and the elderly are traditionally more involved in housework activities that are associated with environmental protection, such as purchasing washing materials, cleaning, classifying garbage/recycling, and so on (Greenbaum, 1995; Tindall et al., 2003; Gong, 2008; Li, 2011; Zhang, 2012).

Second, age groups differ in their pro-environmental behaviors. The young adults and middle-aged groups participate in more organizational pro-environmental behaviors but less on the individual and policy levels. This could possibly occur because younger people have more opportunities to join social group activities than elderly people. The elderly group displays more individual- and policy-level pro-environmental behaviors than the young adults groups, but less involvement in organizational-level pro-environmental behaviors. Such findings are in line with previous studies that have found that the elderly were enthusiastic about household recycling activities (Scott, 1999; Li, 2003) and that the elderly had more opportunities to give opinions at a policy level, due to their status and prestige (Dowd, 1984).

Third, CPC members demonstrate more environmental-friendly behaviors at the individual and policy levels than non-party people. This is consistent with previous studies (Cai et al., 2018) and may be due to the fact that the CPC, as the ruling party, not only often asks members to play a leading/exemplary role in their work and in society, but also holds regular party meetings to disseminate certain policies and information. Therefore, CPC members have a better understanding of environmental problems and related policies than non-members (Tang, 2016; Dong, 2017).

In addition, it is worth noting that we found that the personal monthly income is not statistically significant in relation to individual pro-environmental behaviors, while it has a negative effect on pro-environmental behaviors at the organizational and policy levels. Therefore, the views of “development is an absolute principle” or “treatment after pollution”—which are underpinned by the idea that people would naturally care for environment after the living standards and education levels are improved—are unreliable and even ecologically dangerous assumptions.

In terms of the impact of environmental concerns on people's environmental behaviors, we found that the human domination factor has a significant negative impact on individual- and organization-level pro-environmental behaviors, while the ecological crisis factor has a positive effect on individual pro-environmental behaviors. This is attributed to the idea that anthropocentrists believe that human beings can meet their needs at the cost of their ecological environment. In contrast, residents with an awareness of an ecological crisis worry about the current ecological environment; thus, they will participate in more pro-environmental activities. The dimensions of environmental concern are not statistically significant for policy-level participation; in other words, environmental concerns primarily affect pro-environmental behaviors at the individual and organizational levels, but not at a policy level. We propose two possibilities to explain this result: on the one hand, even though people are more concerned about environmental policy, ordinary people who have never engaged in any environmental movements might not have strong motives to influence policy. On the other hand, to promote environmental governance, policy makers and other stakeholders should provide more convenient channels (e.g., Bulletin Board System; Mobile Applications, etc.) for public participation and encourage people to contribute to environmental policy.

In terms of the relationship between altruism and people's environmental behaviors, we found that altruistic behavior has a positive impact on pro-environmental behaviors, seemingly reflected by the prosocial behavior and social donation factors. Specifically, these two factors have a significant positive impact on individual- and policy-level pro-environmental behaviors; for organizational-level pro-environmental behaviors, only the social donation factor has a significant positive effect, and other dimensions are not statistically significant. This may be due to the fact that social donations are the most easily-accessible environmental activities for people to participate in at the organizational level in mainland China.

In summary, although it seems that the self-interested NIMBY movement is more likely to attract attention (Yang, 2005; Li et al., 2012), our empirical research here shows that altruism and awareness of the ecological crisis can promote people's engagement in pro-environmental behaviors in China. Therefore, it is likely that ENPOs can reach wider audiences of potential supporters and convert more of them into active volunteers. In this way, more people would participate in various environmental activities as a result of education that deepens their awareness in environmental and ecological crises or that advocates and encourages altruism. Meanwhile, the truth of the assumption that people care more for the environment after their living standards have improved is seriously thrown into doubt, because socioeconomic status indicators are not statistically significant for individual-level pro-environmental behaviors.

### Further Research and Limitations

The research that has been undertaken has highlighted a number of topics on which further research would be beneficial. First, though scholars have revealed that environmental knowledge could be an influential factor for environmental behaviors, for the following reasons we did not set out to measure environmental knowledge in this research and instead left the topic to further studies. Firstly, the Chinese scale currently only focuses on pure knowledge of human-ecology systems (e.g., the harm of car exhausts, acid rain, etc.) (Hong and Xiao, 2007). However, based on the literature, we believe that a feasible measurement for this study should also cover knowledge of ENPOs and environmental policies. Secondly, previous research has shown that environmental knowledge is usually highly related to education (Gong and Lei, 2007; Hong and Xiao, 2007; Chen et al., 2011), and demographic data like education is typically much easier to collect than environmental knowledge. Thirdly, we are afraid that it would be too ambitious for us to develop two new scales in one study. Therefore, future studies might, for example, develop a scale to evaluate people's environmental knowledge at scientific (pure knowledge) and social (ENGOs, related policies, etc.) levels, and then the relationships between environmental knowledge and people's environmental behaviors can be further explored.

Second, another interesting field of further research would be the relationships between altruism and donations. For example, as an altruistic behavior across human societies, would the social donation factor and pro-environmental behaviors share similar drives (i.e., motivation or underlying psychological mechanisms)? Moreover, the latest media revolution has led to the new separation between the state and the society; it is worth studying further through interdisciplinary approach to better understand online fundraising, e-donation, and related environmental participations (Xu, 2021).

Third, further exploration is needed into the factors that deter individuals of different groups—such as men, young adults, and the elderly—from engaging in various environmental activities. In particular, due to the higher individual levels of pro-environmental activity participation among women and the elderly, one direction of future study could examine the mediation effect of participating in housework.

Last, but not least, we should acknowledge that there are several research limitations in this study. First, in contrast to other methods such as online surveys, a face-to-face survey can assure a nice level of understanding of the questions. However, the face-to-face survey could possibly raise people's concern about being evaluated by others (e.g., the interviewer), which can, in turn, lead to artificial responses that are more “favorable.” Second, due to limited time and resources, we collected the hardcopy questionnaires in the city in East China and mailed them to a university located thousands of miles away in South China to input the data. As we previously mentioned, the missing data occurred during this study. This may be due to the mailing distance, which led to some answers being difficult to read. In retrospect, it would be better to input the data while at the research site.

## Data Availability Statement

The raw data supporting the conclusions of this article will be made available by the authors, without undue reservation.

## Ethics Statement

The studies involving human participants were reviewed and approved by The Chinese University of Hong Kong. The patients/participants provided their written informed consent to participate in this study.

## Author Contributions

YX conceptualized the theme, collected the data, and wrote the first manuscript draft. SC analyzed the data. WL reviewed and commented on the initial draft. All authors contributed to the article and approved the submitted version.

## Conflict of Interest

The authors declare that the research was conducted in the absence of any commercial or financial relationships that could be construed as a potential conflict of interest.

## Footnotes

^1^The double number of items was assigned for the negative score in the original questionnaire, but we made a corresponding adjustment in the analysis.
